# Mechanical, Rheological, and Bioactivity Properties of Ultra High-Molecular-Weight Polyethylene Bioactive Composites Containing Polyethylene Glycol and Hydroxyapatite

**DOI:** 10.1100/2012/474851

**Published:** 2012-04-30

**Authors:** Mazatusziha Ahmad, Mat Uzir Wahit, Mohammed Rafiq Abdul Kadir, Khairul Zaman Mohd Dahlan

**Affiliations:** ^1^Department of Polymer Engineering, Faculty of Chemical Engineering, Universiti Teknologi Malaysia, Johor, 81310 Skudai, Malaysia; ^2^Department of Biomechanics and Biomedical Materials, Faculty of Biomedical Engineering and Health Science, Universiti Teknologi Malaysia, Johor, 81310 Skudai, Malaysia; ^3^Division of Radiation Processing Technology, Malaysian Nuclear Agency, Selangor, Bangi, 43000 Kajang, Malaysia

## Abstract

Ultrahigh-molecular-weight polyethylene/high-density polyethylene (UHMWPE/HDPE) blends prepared using polyethylene glycol PEG as the processing aid and hydroxyapatite (HA) as the reinforcing filler were found to be highly processable using conventional melt blending technique. It was demonstrated that PEG reduced the melt viscosity of UHMWPE/HDPE blend significantly, thus improving the extrudability. The mechanical and bioactive properties were improved with incorporation of HA. Inclusion of HA from 10 to 50 phr resulted in a progressive increase in flexural strength and modulus of the composites. The strength increment is due to the improvement on surface contact between the irregular shape of HA and polymer matrix by formation of mechanical interlock. The HA particles were homogenously distributed even at higher percentage showed improvement in wetting ability between the polymer matrix and HA. The inclusion of HA enhanced the bioactivity properties of the composite by the formation of calcium phosphate (Ca-P) precipitates on the composite surface as proven from SEM and XRD analysis.

## 1. Introduction

Biomaterials in the form of implant are widely used to replace and/or restore the function of traumatized tissues or organs, to assist in healing, to improve function, and to correct abnormalities [1]. In order to improve the quality of life of patients, design, material selection, and biocompatibility remain as the paramount issues of biomaterials for medical applications. Polymer-based materials are widely used in the medical field, but their utilization in the orthopaedic sector is restricted due to the limitation of their mechanical properties relative to metallic materials. Ultrahigh-molecular-weight polyethylene (UHMWPE) has been used in the orthopaedics mostly as bearing liner due to its low friction and high wear resistance. Extensive work has been carried out by numerous researchers to improve its performance for load bearing applications [2–4]. However, its potential for other biomedical applications can be realized with improved biocompatibility and excellent combination of high strength and wear resistance [5]. For improved biocompatibility, researchers have explored the use of HA and showed its importance for enhanced bioactive and biocompatibility properties [6–8].

The use of UHMWPE as matrix for HA, however, has not achieved much success due to its extremely high viscosity which results in flow resistance and high shear degradation [9] as well as causing difficult processing via conventional techniques. The inability of UHMWPE to flow limits the incorporation of fillers into the plastic matrix to dry mixing only. Furthermore, ensuring HA particles to be well dispersed in UHMWPE matrix has been very challenging [3].

 The development of HA/UHMWPE composites has been extensively studied by Fang and coworkers through solid state mixing, solution gelation and melt compounding methods [2, 3, 10]. Lim et al. [11] prepared the HA composite based on UHMWPE/HDPE matrix using an internal mixer. Although the mechanical properties of UHMWPE/HDPE blend are well documented [12, 13], its processability using polyethylene glycol (PEG) as processing aid has not been investigated and explored.

 Our recent preliminary study found that HDPE with small amounts of poly(ethylene glycol) (PEG) as processing aids reduced viscosity of UHMWPE and effectively improved the processability of UHMWPE without compensating mechanical properties [14]. The inclusion of HA was also reported to progressively enhance the strength of the composites. However, further analysis is required on the effect of HA on mechanical, rheological, and bioactive properties of this composite.

 In this work, HA-reinforced UHMWPE/HDPE/PEG composites were processed using single screw extruder Nanomixer. The use of single screw extruder provides intensive dispersive mixing of fillers and additives with excellent temperature control. Additionally, the unique geometry of the Nanomixer with multiple inlets and outlets can divide, reorient, and recombine the melt stream. The ability of this compounding method allows filler agglomerates to break up into fine particles and disperse thoroughly throughout the polymer melt.

## 2. Experimental

### 2.1. Materials

UHMWPE used was GUR 1020 (Ticona, United Kingdom) and was supplied in a powder form *M*_*w*_ = 3.5 × 10^6^ g/mol with a density of 0.93 g/cm^3^. HDPE (Etilinas HD5403AA, Polyethylenes) was supplied in resin form with a density of 0.954 g/cm^3^ and melt flow rate (MFR) of 0.25 g/10 min. PEG having an average molecular weight of about 8000 was supplied by Sigma Aldrich, Malaysia. HA used was a synthetic calcium phosphate ceramic (Ca_10_(PO)_6_(OH)_2_), grade 21223 (Fluka) manufactured by Sigma Aldrich, Malaysia, with a density of 2.42 g/cm^3^. The specific surface area of the powder, measured by N_2_ absorption (Brunnauer-Emmett-Teller method) was found to be 33.05 m^2^/g. The in vitro test of apatite formation can be reproduced on the surfaces of materials in simulated body fluid (SBF) following the Kokubo method [15]. The SBF was prepared by dissolving reagent NaCl, NaHCO_3_, KCl, K_2_HPO_4_·3H_2_O, MgCl_2_·6H_2_O, CaCl_2_·2H_2_O, and Na_2_SO_4_ into distilled water and buffered with Tris (hydroxyl-methyl-amino-methane, NH_2_C(CH_2_OH)_3_) and hydrochloric acid (HCl) to pH 7.4 at 37 °C. The solution of SBF containing ion concentrations nearly equals to those of human blood plasma.

### 2.2. Sample Preparation

The granules of HDPE were grinded into powder prior to mixing with UHMWPE and PEG. The mixed samples were compounded in a single screw extruder at a screw speed of 80 rpm. The blend and composite formulations are summarized in Table 1. The UHMWPE/HDPE blend system is in wt% with total composition of 100%. In order to investigate the effect of HA content to the UHMWPE/HDPE system, addition of PEG and HA is in per hundred resin (phr) with the UHMWPE/HDPE blend as a basis. The temperature profile of the extruder was set to 195, 220, and 240 °C at the feed zone, metering zone and die zone, respectively, followed with compression molding into samples at 210 °C for 25 min (including 15 min of preheat) under 14 MPa. The test specimen was cut according to ASTM standard. For the purpose of rheological study, the pellets were extruded using capillary rheometer.

### 2.3. Testing

#### 2.3.1. Rheological Test

Rheological measurement was carried out on a capillary rheometer (Gottfert Rheograph 2002) at a temperature of 195 °C. Two round dies with length/diameter (L/D) ratio of 10 and 20 were used. The range of apparent shear rates was between 10 and 2000 s^−1^. The rheological data were calculated directly on the rheometer. No Bagley correction was applied.

#### 2.3.2. Flexural Test

Flexural test was carried out using a Universal Mechanical Testing Machine (Lloyd Instruments) according to the ASTM D790 standard, three-point bending system. A cross-head speed of 5 mm/min was used, and the test was run at a temperature of 25 °C. To retain consistency, a jig that allowed a span of 50 mm was used. The dimension of specimen was 12.7 mm × 3.0 mm × 150.0 mm (width × thickness × length). Five specimens of each composition were tested, and the average values were reported.

#### 2.3.3. Surface Examination and EDX Analysis

The morphology of impact fractured surface of the specimen was observed by field emission scanning electron microscopy (FESEM-Carls Zeiss Supra 35). The study was conducted to examine the distribution of HA particles. The detection and X-ray mapping of elements such as Ca, P, and Si were achieved by using an energy-dispersive X-ray spectrometer (EDX) elemental analysis system (Oxford Instrument, UK). Samples were coated with thin layer of platinum prior to examination under electron beam.

#### 2.3.4. Bioactivity Test

Bioactivity test was performed to determine the ability of HA to bond to bone tissue. Each specimen was immersed in 200 mL of SBF, and the solution was placed in an incubator for 72 hours at temperature of 37 °C. After soaking in the SBF, the specimen was removed from the SBF and dried. Apatite formation on the material surface was examined by scanning electron microscope (SEM) (Philips XL 40).

#### 2.3.5. Characterization of HA

The particle size of HA was measured using Malvern Mastersizer (2000). From this test, the particle size distribution was determined at median particle size (*d *_0.5_), and the sizes below which 10% (*d *_0.1_) and 90% (*d *_0.9_) of the particle diameters lie.

#### 2.3.6. Brunauer-Emmett-Teller (BET)

The particle sizes of HA will be estimated on the basis of the Brunauer-Emmett-Teller (BET) specific surface area using nitrogen as the absorption gas. The particle size will be calculated from BET specific surface area using the empirical equation (1):

(1) $$\;\;\;t=\frac{6}{\rho \cdot S},$$

where *t* is the average grain size in micron, *ρ* the density in g/cm^3^, and *S* the specific surface area in m^2^/g). From the test, the specific surface area was found to be 33.05 m^2^/g. The particle size calculated using the empirical equation is 7.51 *μ*m.

## 3. Results and Discussion

### 3.1. Characterization of HA Particle Size

**Figure 1:**
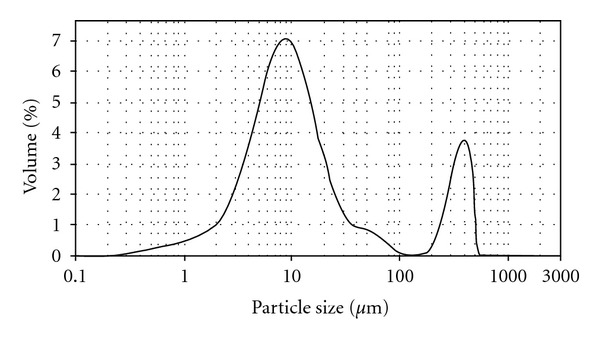
Particle size distribution of HA.

**Figure 2:**
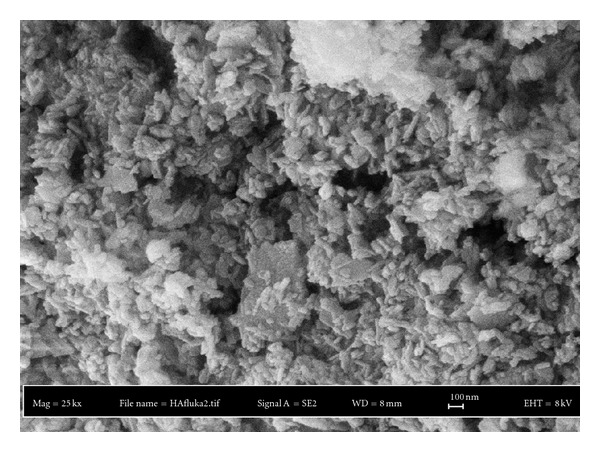
Morphology of HA particles.

**Figure 3:**
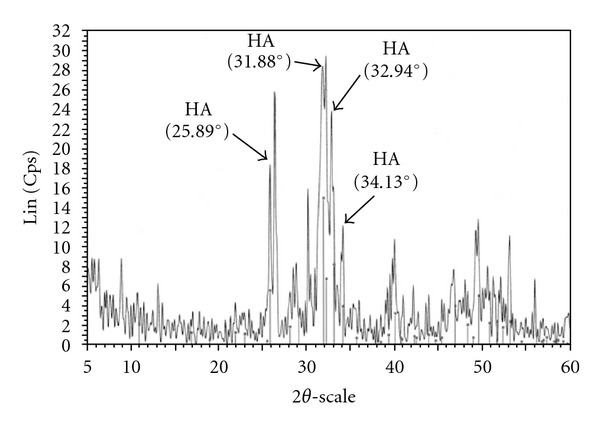
XRD patterns of HA.

Table 2 summarizes the HA particle size below 10%, 50%, and 90% of the particle diameters taken from the particle size distribution result as shown in Figure 1. The result shows a bimodal distribution with the size distribution peaked at 9.36 *μ*m. The median particle size of HA used in this work was 9.99 *μ*m. The largest particle size was approximately 318 *μ*m, while almost 10% of HA particle size was less than 3.19 *μ*m. The morphology of HA particles by SEM examination (Figure 2) shows a nonspherical shape. Similar observations on the morphology of HA were reported by Wang et al. [16] and Joseph et al. [17]. XRD analysis supports the above finding with the powder mainly composed of HA (Figure 3).

### 3.2. Rheological Study: Effect of HDPE and PEG on Viscosity Reduction of UHMWPE

**Figure 4:**
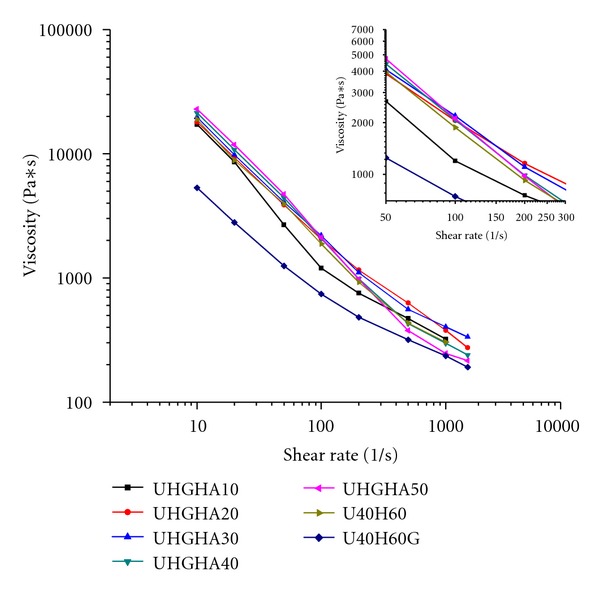
Apparent flow curves of UHMWPE/HDPE, UHMWPE/HDPE/PEG blends, and UHMWPE/HDPE/PEG/HA composites at temperature of 195 °C. The insert shows the curves at shear rates above of 100 s^−1^.

The flow curves of UHMWPE/HDPE and UHMWPE/HDPE/PEG blends and UHMWPE/HDPE/PEG/HA composite at 195 °C are shown in Figure 4. As the processability relates to the resistance of material to processing, the viscosity being the dominant parameter is used to characterize the processability of blends and composites. The data are presented without Bagley correction. Shear rate was varied from 10 to 2000 s^1^. No steady rheology data could be obtained for neat UHMWPE because it was nonextrudable.

Figure 4 shows that UHMWPE/HDPE/PEG blend had the lowest viscosity at all investigated shear rates. Only a slight difference in viscosity value was observed for composites with the variation of HA content. It is clearly observed that melt viscosity of the UHMWPE/HDPE, UHMWPE/HDPE/PEG blends and composites decreased with the increase of shear rate, indicating pseudoplastic behaviour exhibit the non-Newtonian and shear thinning behaviors. Most of the compositions were processable up to apparent shear rate of 2000 s^−1^. As the polymer melt flows, the polymer molecules not only slide past each other, but also tend to uncoil. On release of the deforming stresses, these molecules tend to revert to recoiling. At low shear rates, Brownian motion of the segments occurs, and, therefore, reentanglement is a faster rate than orientation. At high shear rates, the reentanglement rates were slower than the orientation rates, thus resulted in less viscous polymers [18], and this reasons supports the reduction of polymer viscosity at high shear rate which is several orders of magnitude smaller than the viscosity at low shear rates. Although the viscosity of the UHMWPE/HDPE blends was lower at high shear rate, the processability of UHMWPE/HDPE blends with high content of UHMWPE was reduced. These results were expected due to the high amount of UHMWPE which has high-molecular-weight and has longer reentangling and relaxation time. Thus, limits the processable value to the lower shear rate.

Incorporation of PEG decreased the UHMWPE/HDPE blends viscosity significantly. The blends showed a noticeable difference in viscosity at all shear rates. The rapid decrease of viscosity was probably due to the alignment of PEG chains in neighbouring molecules of polyethylene blends, increased the movement of polymer chain, and therefore reduced the viscosity. The stronger the interchain interactions between different polymers reduced the tendency for entanglement. On contrary, UHMWPE and HDPE chains have high potential to entangle with each other than different polymer, thus contribute to high viscosity. Interchain interactions influenced the mobility of macromolecules and such processes as interdiffusion and phase separation [19]. Flow properties of polymer blend mainly depend on the compatibility between the blend components [20], and the molecular compatibility of polymers components is effected by interchain interactions. Thus, it is believed that interchain interactions are the main factor influencing the results obtained. Our results reflect the obtained synergistic effects by combining PEG with HDPE. PEG acts successfully as an internal lubrication due to the good lubrication characteristics and promotes the interphase slippage of the blend. SEM results support the evidence of the existence of PEG in the interior of samples.

 The influence of PEG on the reductions of UHMWPE blends viscosity was also reported by Xie et al. [21] who proposed that the mechanism of viscosity reductions of UHMWPE/PP blend was contributed by two factors, internal lubrication in the melt and external lubrication of extrudate on the die wall surface. They concluded that the driving force for the external lubrication came from two aspects. The first is the higher surface energy of PEG than UHMWPE and PP which tend to migrate to the die wall surface. The second is due to the lower viscosity of PEG compared to UHMWPE and PP that easily migrate to the skin layer of the melt where the shear rate is maximum. According to Naranjo et al. [22], surface tension between two materials appears as a result of different intermolecular interactions and was found to play a significant role in the deformation of polymers during flow, especially in dispersive mixing of polymer blends. Based on this paper, a similar factor is expected to contribute in the melt viscosity reductions of UHMWPE/HDPE/PEG blends. When the migration of PEG to the die wall surface occurs, it is reasonable to observe an apparent viscosity and shear stress reduction for UHMWPE/HDPE/PEG blend as a result of melt slippage on die wall. The surface tension values of UHMWPE and PEG are 26.16 and 28.46 mN/m, respectively [21].

 Inclusion of HA displays significant increment in viscosity relative to the viscosity of UHMWPE/HDPE/PEG blends at any given shear rate. However, only a slight difference in viscosity was observed between the composites and UHMWPE/HDPE blend. The viscosity increment with addition of 10 phr HA at low shear rate below 100 s^−1^  was about twofold higher than the UHMWPE/HDPE/PEG blends. The composites viscosity increased by 33 to 76% with the increase of HA content from 10 to 50 phr, respectively, in the range of shear rate up to 50 s^−1^. Viscosity of the composites tends to increase systematically with increasing of HA loading from 10 to 50 phr at shear rate below 100 s^−1^. The result obtained is expected as it is well known that addition of rigid filler results in more viscous polymer, especially when a physical network is formed. The increment in composites viscosity is in similar trend up to 100 s^−1^. Interestingly, at shear rate of 100 s^−1^, all the composites viscosity reached almost the same value as shown by the zoom insert in Figure 4. It can be noted that the point of 100 s^−1^ seemed to be the critical point of the composites viscosity. It is worthy to observe that above this critical point, as the shear rate increased up to 1500 s^−1^, a reverse trend is observed in composites viscosity. Apparently, a composite with 50 phr HA shows the lowest viscosity compared to those composites, and even lower than UHMWPE/HDPE blends. The highest viscosity is observed for composites at 30 phr HA. However, composites with 10 phr HA obviously show a different manner which closely resemble the curves of UHMWPE/HDPE/PEG blend. Shear viscosity of the UHMWPE/HDPE/PEG/HA composites is a decreasing function of shear rate and increasing function with HA loading which are in agreement with the findings reported by Wang et al. [16]; however, it is valid at shear rate below 100 s^−1^  only. At the point of below 100 s^−1^, it is reasonable to assume that the reentanglement rate is faster than the orientation rate. Thus, the presence of PEG has no contribution in assisting filler orientation to follow the direction of applied shear stress. Therefore, the viscosity of the composites increases with increasing HA content as addition of filler hinders the alignment of polymers chain in flow direction. At high shear rates above 100 s^−1^, the orientation rates are faster than the reentanglement rates thus result in less viscous polymers. A significant effect of PEG was observed at this stage. It is proposed that the HA was coated with PEG as it is well known that PEG having low friction coefficient, low molecular weight, and partial miscibility with UHMWPE and HDPE has a high tendency to coat the HA particles. The combination of these two factors provides easier movement of HA particles to slip into the matrix, improved the filler orientation and ordering with flow. Specifically in the case of composite at 50 phr HA, more HA particles dispersed in fine size as observed from SEM results, indicating more HA with larger surface area was coated with PEG, therefore improved resistance to flow and decrease the viscosity. However, as explained by Joseph et al. [17], HA with larger surface area required more matrix to wet the surface area and reduced the effective volume of HDPE matrix to promote shear flow between HA particles. In the present case, it is strongly believed that when HA was coated by PEG, less PE matrix was required to wet the filler surface. In other words, the volume of PE matrix increased and remained available to promote shear flow, hence, reducing the viscosity of the melt. In contrast, the agglomeration of HA particle in large size reduces the part of PE matrix volume available for shearing during polymer flow. This can be further confirmed by morphological analysis showing that the presence of larger HA agglomerates in composites of 30 phr HA results in the highest melt viscosity, followed by 20 phr HA. Yang et al. [23], in their study on the effects of PEG molecular weights on rheological behavior of alumina injection molding feedstocks, claimed that the decrease in viscosity with increasing shear rate may reflect improved homogeneity. The potential of PEG in reducing the viscosity of composites was also reported by Sun and Li [24], who investigated the effectiveness of intercalated montmorillonite (MMT)/PEG binary processing aids in reducing the melt viscosity of metallocene linear low-density polyethylene (mLLDPE). They reported that PEG reduced the friction between MMT and matrix by coating the MMT layers and helping polymer chains to unwind.

### 3.3. Flexural Strength

**Figure 5:**
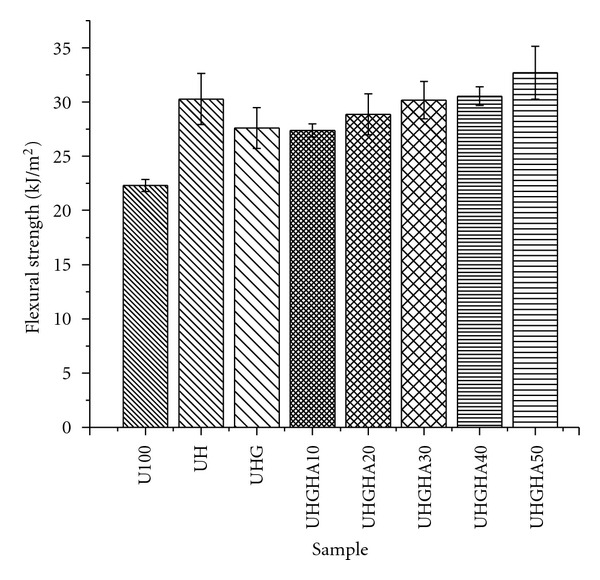
Flexural strength of UHMWPE/HDPE, UHMWPE/HDPE/PEG blends and UHMWPE/HDPE/PEG/HA composites.

Flexural strength of UHMWPE, UHMWPE/HDPE, and UHMWPE/HDPE/PEG blends together with UHMWPE/HDPE/PEG/HA composites is shown in Figure 5. In general, there was a progressive increase in flexural strength of UHMWPE up to 36% with addition of HDPE. The flexural strength of UHMWPE/HDPE/PEG blends was found to decrease with the incorporation of PEG. Interestingly, it can be observed that the composites exhibit a significant increase in flexural strength with inclusion of HA up to 50 phr. The strength increment of the composites was about 18% higher than the UHMWPE/HDPE/PEG blends with the strength value initially maintained at 10 phr HA loadings.

 The possibility of increment in flexural strength at 60 wt% HDPE content for UHMWPE/HDPE blends might be due to less entanglement between polymer chains as the amount of UHMWPE was reduced. Therefore, the restriction of chain movement was reduced and a higher stress needed to allow the slippage of polymer chains before the rupture occurs. It is believed that a higher stress needed is an indication of good interaction between HDPE and UHMWPE. This is in agreement with Mohanty and Nando [25] who reported that a good interaction between the blend components is one factor that leads to a synergistic effect. These can be responsible for the observed mechanical behavior.

**Figure 6:**
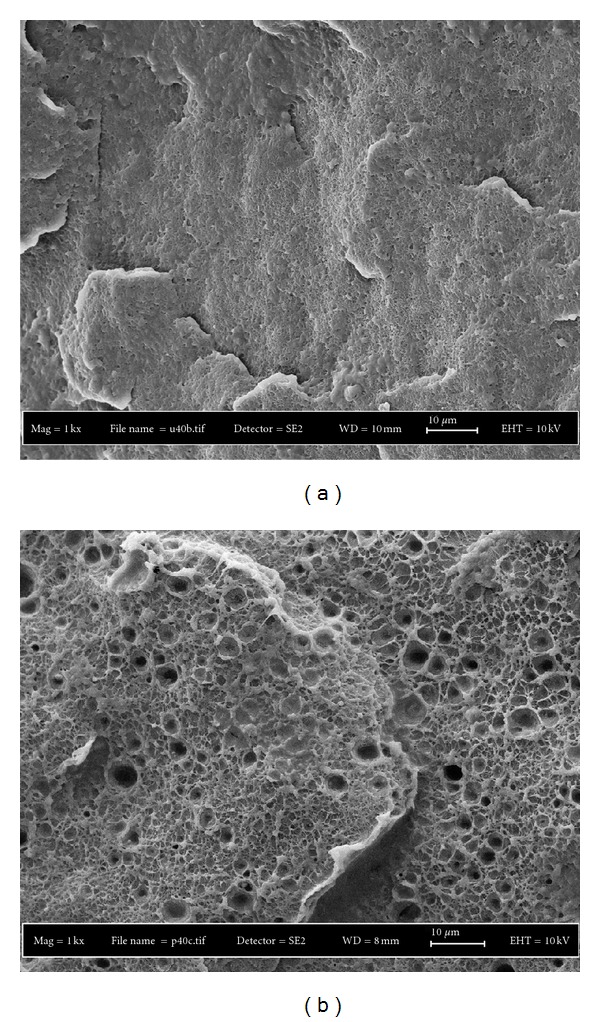
Fracture surfaces of (a) UHMWPE/HDPE (40/60) blend and (b) UHMWPE/HDPE (40/60) blend containing 2 phr PEG.

A lower strength observed for the blends containing PEG is attributed to poor adhesion between the matrix and PEG indicated by the formation of a dispersed PEG in UHMWPE/HDPE matrix. PEG act as a dispersed phase because of the low viscosity of PEG which in turn produced immiscible blends. Thus, lowered the ability of the polymer to sustain higher stress, and dropped the strength values.  Figure 6 shows the SEM result of blends containing PEG.

 A factor which influenced the strength of the composites is the effect of HA on the crystallization kinetics of the matrix. For the present case, the variation in mechanical properties upon particulate filling is not only attributed to interfacial interaction but also by dissimilarities in the semicrystalline structure. This reason supports the maintained strength at 10 phr HA. At this composition a well dispersed HA particles with no agglomerations, providing more site for nucleation and crystal growth. The stiffening effect of the HA particles may be overlapped by the consequence of matrix crystallinity modifications.

 Sousa et al. [26] described the importance of this factor for the interpretation of mechanical performance variation of HA-filled HDPE composites. They reported that mineral particles can act as nucleating agents of polymer matrix, which may affect the semicrystalline structure of the polymer matrix and consequently the mechanical properties of the composite. They also highlighted that this factor is relevant for composite with low filler amounts. Hence, such effect is possible to expect for composites at 10 phr HA which results in marginal increase in flexural strength although the filler particles are well distributed.

The gradual increased with increasing HA content indicates an improvement in mechanical bonding between fillers and polymer matrix due to the homogenous distribution of HA in the polymer matrix. Thus, it is believed that the wetting ability of the matrix to the surface area of fillers was enhanced, entrapped the HA particles in the matrix, and enable more stress to be transferred from the matrix to the fillers. This subsequently prevents filler debonding to fracture at lower strength. This was in agreement with several study reported on HA-filled polyethylene composites where the strengthening effects of HA is highly influenced by the filler dispersion in polyethylene matrix and the wetting ability of the matrix to filler particles [3, 8, 15, 27]. The amount of surface contact between the polymer matrix and the filler can be determined by the specific surface area as it determined the interfacial interaction which significantly affects the mechanical properties of the composites. Fillers with higher surface areas will contribute to more surface contact between the filler and matrix, thus increasing the mechanical properties of the composite [28]. In this work, the specific surface area of HA is 33.05 m²/g (from particle size distribution data) with its irregular shape showing encouraging result and is favorable to improve surface contact between the filler and matrix, thereby increasing the strength of the composites. This type of irregular shape is preferred to the spherical shape, as the molten polymer can penetrate into troughs on the particle surface during high-temperature composite processing and thus form mechanical interlock with the particle even in the absence of chemical bonding [29]. The mechanical interlocking of HA particles by the matrix developed upon the shrinkage of the polymer during cooling. Hence, the composite exhibits a stronger resistance to deformation that made the fracture of the composite to occur higher than the ultimate strength of UHMWPE blends. The presence of agglomerate, however, limits the composites to achieve higher strength up to the desired values. Agglomeration of HA particles in the composite may act as stress concentration points or points of discontinuity in the composites. As a result, the composites have poor interfacial adhesion between matrix (organic) and filler (inorganic) and causing cracks at the particle-matrix interface and particle-particle interface. When stress is applied, the HA fillers have poor capability to support stress transmitted from the matrix and act as crack initiation. With a further introduction of stress to the system, the incapability of HA particles to support the transfer of stress from the matrix subsequently leads to crack propagation and finally results in brittle failure, thereby causing an early fracture of the composites. Wang [29] reported that agglomeration of HA particles either in the condensed state or the intermediate state may not be sufficient to improve the mechanical properties. A slight decrease in flexural strength at low HA loading was not surprising since other studies have also indicated that the incorporation of filler into thermoplastic matrix may not necessarily increase the tensile strength of the composites [8]. The trend obtained is in agreement with study reported by Ramírez et al. [27] who worked on HA as filler in polymer composite. The flexural strength results clearly indicate that polymer crystallinity modifications and filler orientation with homogenous distribution of fine HA particles are two dominating factors that influenced the strength of the UHMWPE composites at low and high HA loadings, respectively.

### 3.4. Flexural Modulus

**Figure 7:**
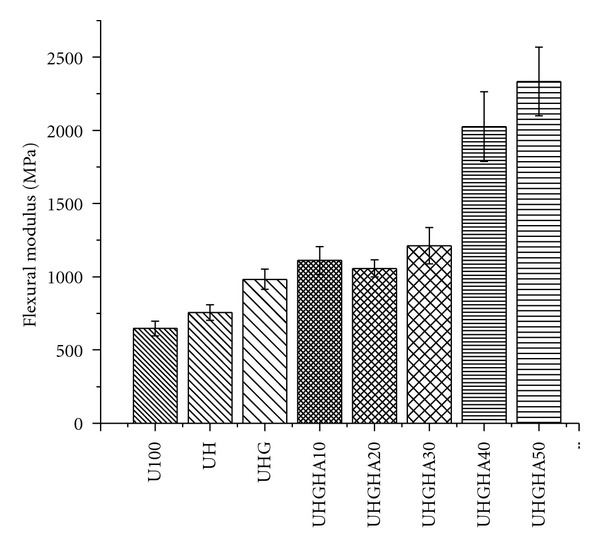
Flexural modulus of UHMWPE/HDPE, UHMWPE/HDPE/PEG blends, and UHMWPE/HDPE/PEG/HA composites.

The flexural modulus of UHMWPE, UHMWPE/HDPE, UHMWPE/HDPE/PEG blends, and UHMWPE/HDPE/PEG/HA composites are shown in Figure 7. It clearly shows that the flexural modulus increased systematically with addition of HDPE, PEG, and HA as compared to the neat UHMWPE. Incorporation of HA results in a significant improvement in flexural modulus of UHMWPE composites. Further increased in modulus was observed with increasing HA content. The increment of modulus was about 92% higher than the UHMWPE/HDPE/PEG as the HA content increased from 30 to 50 phr.

 It is an expected behaviour that the addition of HDPE enhances the stiffness of the blends thus increased the flexural modulus. A similar finding was reported by Nugay and Tinçer [30] in their study on the blends of LDPE/HDPE where the addition of HDPE enhances the modulus to strain hardening increase. It has been proven that the occurrence of the rigid amorphous phase in the case of low-molecular-weight polyethylene gives a higher stress value during stretching [31].

The addition of PEG increased the modulus of UHMWPE/HDPE/PEG blend that can be attributed to the effect of PEG which is mainly located in the amorphous region of the blends. It is believed that the combination of HDPE and PEG chains in the amorphous part has a high tendency to produce a rigid amorphous phase. With a further introduction of stress to the system, the amorphous regions would follow the deformation which subsequently leads to an orientation of the amorphous regions. The system with rigid amorphous phase therefore results in increment of elastic modulus although the degree of crystallinity is reduced. This is reasonable since the previous study by Xie and Li [32] also found the role of PEG additives in the amorphous phase.

The modulus of the composites shows that HA was effectively reinforced by the UHMWPE/HDPE/PEG blends and demonstrates the stiffening effect of HA particles. The increment of modulus is expected, since HA has a higher modulus than the polymer, and a similar trend observed also reported by other studies [3, 33]. The role of HA fillers as the major load bearing component of bone was found in the elastic region rather than the plastic region [34]. The trend is consistent with previous studies who investigated the effect of HA on PE composites [8]. The modulus of the composite that was determined before any significant plastic deformation takes place has only a very weak dependence on the specific surface area and particle shape of the fillers [28]. Thus, it is believed that the increase in the modulus is due to the HA particles which are sufficiently wetted by the polymer surrounding it, restricting the mobility and deformability of the matrix by hindering the movement of polymer molecules.

### 3.5. Bioactivity Properties

**Figure 8:**
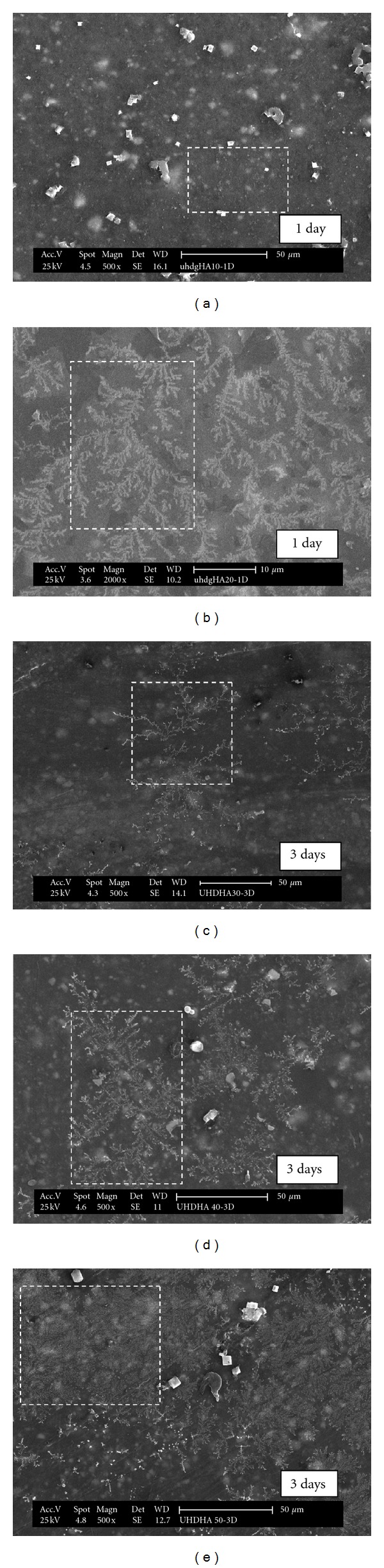
Apatite formation on UHMWPE/HDPE/PEG/HA composites for (a) 10, (b) 20 (c) 30, (d) 40, and (e) 50 phr HA content. The dashed box shows the area of the element analysis.

**Figure 9:**
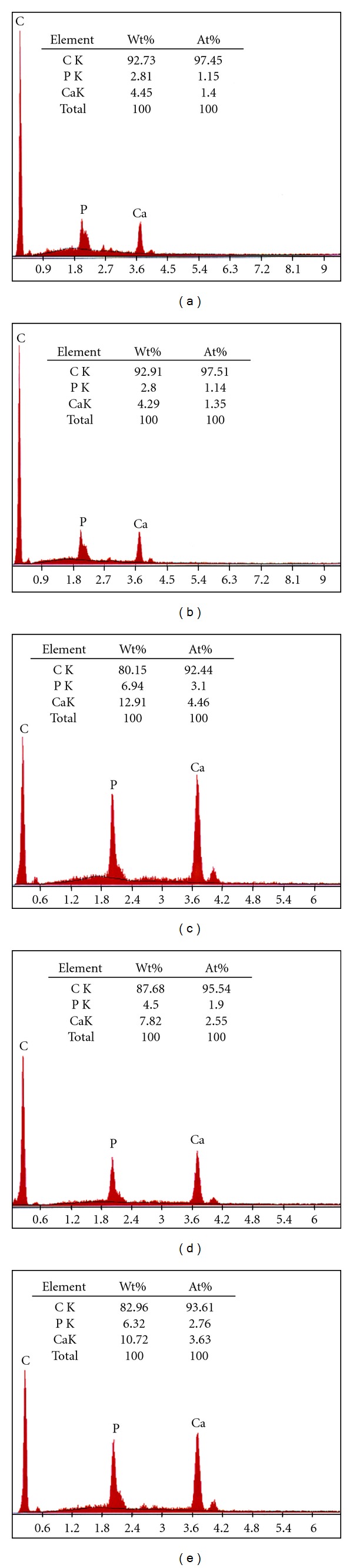
EDX analysis of UHMWPE/HDPE/PEG//HA composites for (a) 10, (b) 20 (c) 30, (d) 40, and (e) 50 phr HA content.

In this study, material bioactivity is assessed by evaluating the ability of the composites to induce carbonate apatite formation on the composites surface. The SEM micrographs accompanied by the EDX spectrum (Figure 9) of the composites surface after the immersion in simulated body fluid (SBF) solutions for 1 to 3 days are shown in Figure 8. The initial formation of apatite layers, a “tree-branching” like, is clearly observed in Figure 8(b) to 8(e) for composites with 20 to 50 phr HA content, respectively. However, there was no formation of apatite layer (calcium-phosphate) (Ca-P) layer for composite at 10 phr HA loadings confirming the bioinert properties similar to PE as shown in Figure 8(a). It is interesting to observe that as the amount of HA increases, the growth of apatite crystal almost completely covered the entire composite. Specifically, composite at 50 phr HA (Figure 8(e)) obviously shows a rapid growth of bulk formation of thicker apatite layer covering the composites surface uniformly, an evidence of excellent bioactivity properties of HA. It should be noted that the immersion time of apatite crystal was observed as early as 1 day. The results confirmed the occurrence of biological response at the interface of the composite caused by the ion exchange reaction during immersion. In this study, composite with HA content less than 20 phr shows that no deposition of apatite layer. Fang [35] investigated on bioactivity properties of UHMWPE/HA composite found that a minimum concentration of HA in the composite to induce the Ca-P layer is less than 30%. Another study reported by Espigares et al. [7] confirmed that PE composites with HA less than 20% behave like bioinert material with no apatite layer formed. Thus, it can be concluded that the minimum HA concentration to induce apatite layer of UHMWPE/HDPE/PEG composites is 20 phr.

 In vitro carbonite apatite layer formation on Ca-P-based materials in SBF is a precipitation process as evidenced by the consuming of calcium (Ca^2+^) and phosphate (HPO_4_^2−^, PO_4_^3^) ions from the SBF. The amount of carbonate apatite formed on the composite is directly influenced by solubility of the HA used. It was reported that the comparison on the population of carbonate apatite on the surface between synthetic HA and coralline HA shows that much more carbonate apatite layer was observed for coralline HA compared to synthetic HA due to its higher solubility which is associated to the smaller size of HA [36]. Based on this observation, the results obtained are reasonable where HA with the size of 7.51 *μ*m produced a uniform apatite formation which is comparable to previous findings [7, 35]. The morphological result of composite surfaces supports the results obtained. Composites of 50 phr HA with homogenous dispersion of HA in polymer matrix is an advantage too, to ensure the fast growth and uniform formation of apatite on the composites surfaces. It is believed that the apatite nucleated initially on the rich part of HA especially for the area with agglomeration of HA. The smaller size of HA provides more surface area that can be exposed to the SBF solution which therefore enhanced the bioactive surface area to act as nucleation sites to induce apatite layer. Due to that, the apatite layer continuously spreads and covers the bioinert polymer area of the composites. This is in agreement with the findings reported by Fang [35] who concluded that the bioactivity properties were controlled by the surface area of HA particles exposed to the physiological environment. The determination of these properties to the composites developed is critically important in the elimination of scaffold loosening [37]. It clearly indicates that the surfaces of UHMWPE/HDPE/HA bioactive composites are compatible to bone growth with the apatite providing a bond between the tissues and composites.

 EDX analysis, performed on the surface of the immersed composite samples, reveals the presence of Ca and P elements on the surface of the specimens. The strong peak for carbon (C) indicates a large amount of carbon on the surface possibly derived partly from the solution. The atomic percentage of Ca and P was more intense with increasing HA content. The Ca/P ratios range between 1.53 and 1.86, that is, between tricalcium-phosphate and tetracalcium phosphate. It is clearly recognized that the apatite layer is equivalent to the mineral in bone both structurally and chemically [38] although the idealized mineral in bones is hydroxyapatite [36]. According to Rea et al. [39], formation of apatite crystals is accompanied with a decrease of C and increase in Ca and P content which reflects that the calcium phosphate layer covered the PE surface. A similar finding is observed in this study indicating apatite deposition on the composite surface increases with increasing HA content.

### 3.6. Morphology and XRD Analysis of UHMWPE/HDPE/HA Composites

SEM analysis was performed in order to study the dispersion of HA particles in the polymer matrix and the effect of PEG on the morphology of UHMWPE/HDPE blends. Comparison on the fracture surfaces of UHMWPE/HDPE and UHMWPE/HDPE/PEG blends (Figure 6) shows that PEG forms dispersed particles since it is incompatible with UHMWPE. SEM micrograph shows that a lot of holes that resulted from the pulling out of the dispersed phase during the fracture are due to PEG dispersed particles. The existence of PEG in the interior of samples indicates that PEG acts successfully as internal lubrication.

**Figure 10:**
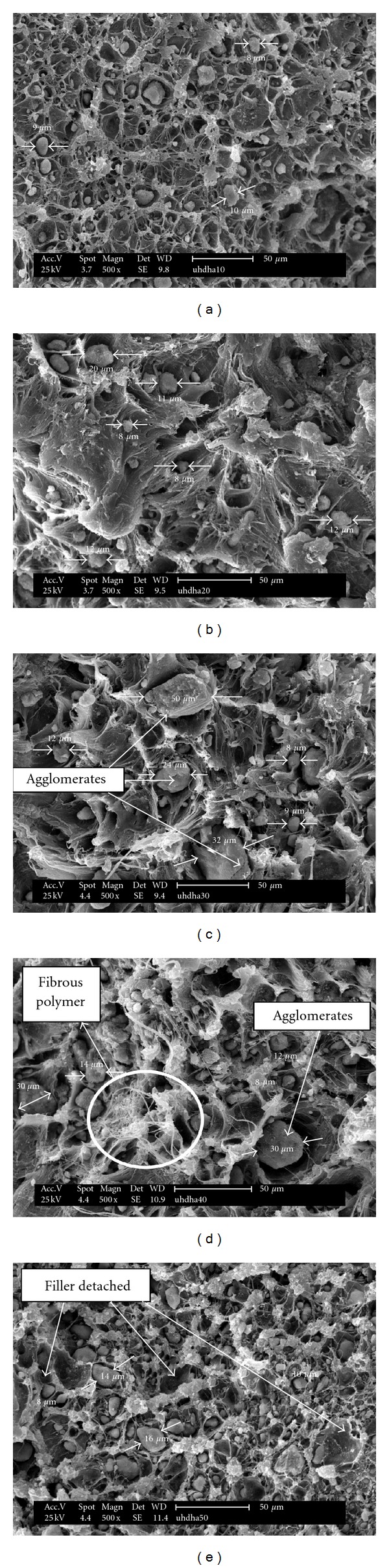
Fracture surfaces of UHMWPE/HDPE/PEG composites for (a) 10, (b) 20 (c) 30, (d) 40, and (e) 50 phr HA content.

**Figure 11:**
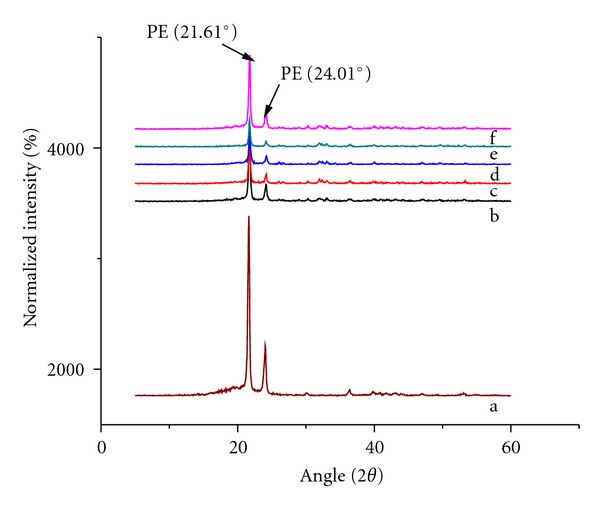
XRD patterns of (a) UHMWPE/HDPE/PEG blend, (b) 10, (c) 20, (d) 30, (e) 40, and (f) 50 phr HA content.

Figure 10 shows the micrograph of UHMWPE composites containing 10 to 50 phr HA content. The SEM micrographs show a two-phase morphology for all the UHMWPE composites systems. The shrinkage of polymer matrix around individual HA particles during cooling cause the formation of mechanical bonding. It is observed that at 10 phr HA, the dispersed particles are uniformly distributed with the HA size less than 10 *μ*m. No agglomerates are found in the morphology since the particle size of HA used is 7.51 *μ*m. Due to low amount of HA, the fillers are well dispersed in polymer matrix although the HA tends to combine and form aggregates. However, composites containing HA from 20 to 40 phr show only a few exposed of HA particles. Some of the HA particles are found to agglomerate and become completely embedded in the polymer matrix as indicated by the arrows in Figures 11(c) to 11(d). Obviously, the largest agglomerate size was found at composites of 30 phr HA which creates more discontinuous sites in the polymer matrix and acts as a failure point. The fibrous polymer strands formed clearly indicate the capability of matrix to sustain further deformation after debonding of HA takes place. At these compositions, the efficiency of HA dispersion in polymer matrix were decreased. The heterogeneity of the material was higher with the increase of HA content. Further, it can be observed that the size of agglomerates decreased with increasing HA content accompanied by the increasing amount of smaller HA particles. On contrary, as the HA content increase up to 50 phr HA, a homogenous distribution of HA particles is observed contributing to the excellent mechanical and bioactivity properties of the composites. The trend of the composite morphology is possibly due to the bimodal particle size distribution, where the small HA particles occupy the space between large particles leading to high HA content with smaller size per unit volume in the composite. In addition, the size distribution peaked at 9.36 *μ*m implying that a lot of a smaller HA particles dispersed in polymer matrix. Failure of the composites is evident by the hole caused by detachment of the HA particles from the polymer matrix as shown by the arrows (Figure 10(e)).

**Figure 12:**
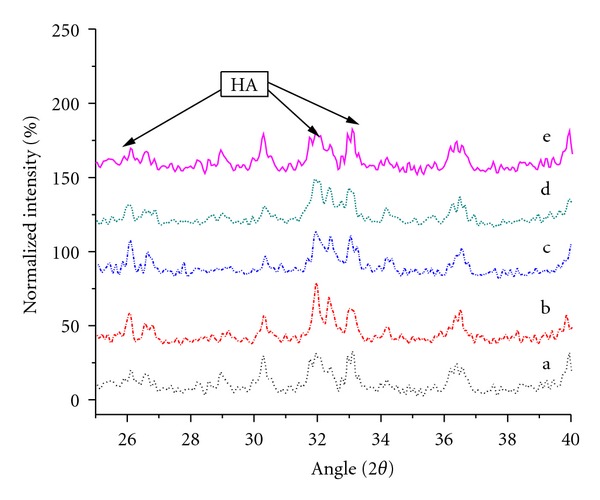
XRD patterns of UHMWPE/HDPE/PEG composites for (2*θ* range from 25 to 40) (a) 10, (b) 20 (c) 30, (d) 40, and (e) 50 phr HA content.

The presence of HA on the composites is further confirmed by XRD patterns as shown in Figures 11 and 12.  Figure 11 shows both the UHMWPE and HDPE peaks are very strong and sharp with very high intensity. Inclusion of 10 phr HA loading reduces the intensity of both PE peaks, which can be attributed to modification of matrix crystallinity. However, the intensity of PE peaks was clearly increased with increasing HA content indicating the effectiveness of HA as nucleating agents in the composites. It is highly possible that HA modifies the amount of crystalline phases in the composites and subsequently affects the mechanical properties of the composites as claimed in the previous section. The exaggerated HA characteristic peaks from Figure 11 are shown in Figure 12. The typical peaks of HA in the composite were almost similar with the HA powder as shown in Figure 3. No remarkable change in the peak intensity is observed with increasing HA loadings. The band intensity of HA peaks is sharp for the whole investigated composites. This analysis clearly demonstrates a strong relationship between the mechanical properties and bioactivity properties of the composites with respect to the changes in the morphologies and composite structure.

## 4. Conclusions

The viscosity of UHMWPE/HDPE/PEG blends is reduced significantly with the incorporation of PEG. Good interaction between the UHMWPE/HDPE blend components is one factor that leads to a synergistic effect and contributes to strength enhancement. Incorporation of HA results in improvement of modulus and strength of the composites with increasing filler concentration. The changes in composite morphologies show a strong relation with the mechanical properties and bioactivity properties of the composites. In addition, the changes in structure of the composites analyzed by XRD further confirmed the role of HA as nucleating agents which in turn modify and influence the mechanical properties of the composites. SEM micrographs reveal the formation of the apatite layer covering the composites surface that contributes to bioactive properties. Overall, the viscosity of UHMWPE is successfully reduced to a processable value with improvement in mechanical properties of the composites which are comparable to the cancellous bone properties and is preferable in biomedical applications.

## Figures and Tables

**Table 1:** Composition of the UHMWPE composites.

Designation	UHMWPE	HDPE	PEG	HA
	(wt%)	(wt%)	(phr)	(phr)
U100	100	0	0	0
UH	40	60	0	0
UHG	40	60	2	0
UHGHA10	40	60	2	10
UHGHA20	40	60	2	20
UHGHA30	40	60	2	30
UHGHA40	40	60	2	40
UHGHA50	40	60	2	50

**Table 2:** Particle size of HA.

Sample	Particle size (*μ*m)			Specific surface area (m^2^/g)
	*d*_0.1_	*d*_0.5_	*d*_0.9_	
HA	3.19	9.99	318.47	33.05
